# Philosophy of transcendental cinema and its applications in psychiatry. The case of lost highway by david lynch

**DOI:** 10.1192/j.eurpsy.2024.1374

**Published:** 2024-08-27

**Authors:** J. K. Nowocień, N. Szejko

**Affiliations:** Department of Medical Ethics and Palliative Medicine, Medical University of Warsaw, Warsaw, Poland

## Abstract

**Introduction:**

Thanks to Paul Schrader, transcendental cinema was distinguished from the slow cinema trend. What distinguishes it from it are the precise psychological portraits of the characters and the aptly reproduced world of internal experiences. Transcendental cinema draws from the philosophy of existentialism, presenting the assumptions of the human psyche. In D. Lynch’s cinematography, we can find faithful representations of mental disorders, such as dissociative fugue, depersonalization, mania or psychosis. Based on the “Lost Highway” (1997), we will prove that D. Lynch, with his cinematography, not only provides knowledge about mental disorders, but also gives patients humanity and dignity. The series also resembles a meditation session in the style of mindfulness, which, when practiced, helps a person affected by mental illness in his recovery process.

**Objectives:**

The aim of this work is to indicate the accurate record of the inner characters’ experiences in D. Lynch’s cinematography, which provides us with knowledge about mental disorders of an individual. By creating a visual image that affects many senses, transcendental cinema sensitizes us and makes us aware of the suffering of a patient affected by mental disorders. The session, while drawing on the philosophy of mindfulness, becomes a meditative session, therapeutic for both us and the protagonist.

**Methods:**

In this research we use the approach proposed by Paul Schrader and David Lynch to analyze analyze transcendental cinema as an art that combines philosophy, cinematography and psychiatry. As a representation of the experiences of a person outgoing the therapy basen on psychoanalysis.

**Results:**

Many studies indicate the positive impact of mindfulness meditation on physical and mental health. Through long scenes, transcendental cinema draws attention to individual stimuli reaching our body, non-judgmental noticing them, focusing on one thought and one sensation, draws from the philosophy of mindfulness, becoming a meditative session in itself. Therefore, a film screening provides us with knowledge about the internal experiences of a psychiatric patient, indicates the form of therapy and at the same time leads us through a therapeutic meditation session.

**Conclusions:**

We believe that the transcendental cinema represented by David Lynch can be treated not only as a representation of mental disorders and the suffering associated with them, but also as a meditative, healing and liberating session. Not only for the person affected by the disorder, but also for us as viewers.

**Disclosure of Interest:**

None Declared

